# Haplotyping and copy number estimation of the highly polymorphic human beta-defensin locus on 8p23 by 454 amplicon sequencing

**DOI:** 10.1186/1471-2164-11-252

**Published:** 2010-04-19

**Authors:** Stefan Taudien, Marco Groth, Klaus Huse, Andreas Petzold, Karol Szafranski, Jochen Hampe, Philip Rosenstiel, Stefan Schreiber, Matthias Platzer

**Affiliations:** 1 Leibniz Institute for Age Research - Fritz Lipmann Institute, D-07745 Jena, Germany; 2 Institute of Clinical Molecular Biology, Christian-Albrechts-University D-24105 Kiel, Germany; 3 Dept. of General Intermal Medicine, Christian-Albrechts-University D-24105 Kiel, Germany

## Abstract

**Background:**

The beta-defensin gene cluster (DEFB) at chromosome 8p23.1 is one of the most copy number (CN) variable regions of the human genome. Whereas individual DEFB CNs have been suggested as independent genetic risk factors for several diseases (e.g. psoriasis and Crohn's disease), the role of multisite sequence variations (MSV) is less well understood and to date has only been reported for prostate cancer. Simultaneous assessment of MSVs and CNs can be achieved by PCR, cloning and Sanger sequencing, however, these methods are labour and cost intensive as well as prone to methodological bias introduced by bacterial cloning. Here, we demonstrate that amplicon sequencing of pooled individual PCR products by the 454 technology allows in-depth determination of MSV haplotypes and estimation of DEFB CNs in parallel.

**Results:**

Six PCR products spread over ~87 kb of DEFB and harbouring 24 known MSVs were amplified from 11 DNA samples, pooled and sequenced on a Roche 454 GS FLX sequencer. From ~142,000 reads, ~120,000 haplotype calls (HC) were inferred that identified 22 haplotypes ranging from 2 to 7 per amplicon. In addition to the 24 known MSVs, two additional sequence variations were detected. Minimal CNs were estimated from the ratio of HCs and compared to absolute CNs determined by alternative methods. Concordance in CNs was found for 7 samples, the CNs differed by one in 2 samples and the estimated minimal CN was half of the absolute in one sample. For 7 samples and 2 amplicons, the 454 haplotyping results were compared to those by cloning/Sanger sequencing. Intrinsic problems related to chimera formation during PCR and differences between haplotyping by 454 and cloning/Sanger sequencing are discussed.

**Conclusion:**

Deep amplicon sequencing using the 454 technology yield thousands of HCs per amplicon for an affordable price and may represent an effective method for parallel haplotyping and CN estimation in small to medium-sized cohorts. The obtained haplotypes represent a valuable resource to facilitate further studies of the biomedical impact of highly CN variable loci such as the beta-defensin locus.

## Background

Since the pioneering publication of Margulies et al. [1] many researchers have demonstrated the versatility of the massively parallel 454 pyrosequencing technology. The method has been successfully applied to a large diversity of targets such as nuclear and organellar genomes [2-4], transcriptomes [5-8], cloned DNA (e.g. BACs) [9], and PCR products (amplicons) [10]. This broad spectrum is complemented by a wide range of analyses, including epigenetic features [11,12], genome diversity [13,14] and ancient DNA [15].

**Figure 1:**
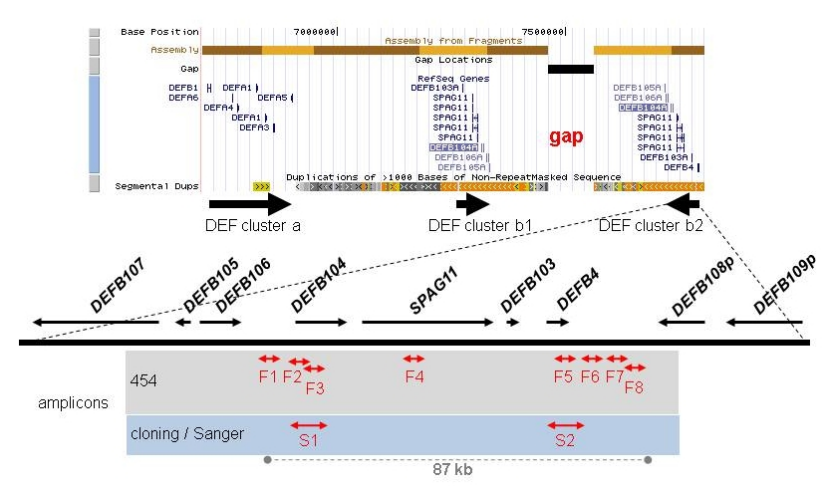
**Human defensin gene clusters at 8p23.1 (NCBI build 36, hg18) and location of the amplicons analyzed by 454 (F1-8) and cloning/Sanger (S1-2) sequencing within the proximal DEFB (DEF cluster b2)**.

In principle, the 454 technology should also be applicable for haplotyping copy number (CN) variable loci such as the beta-defensin gene cluster (DEFB) at human chromosome 8p23.1 (Fig. 1). This locus is intensively studied [16-26] is recognized as one of the most dynamic regions of the human genome [27] and was proven to be variable in CN as a whole [28]. Individual DEFB CNs and specific DEFB haplotypes have been shown to be associated with psoriasis, Crohn's disease and prostate cancer [18,20,29,30]. Within the 2 DEFBs assembled in the human reference sequence (234 and 224 kb; hg18, build 36.1, chr8:7,156,778-7,391,276 and chr8:7,669,629-7,893,454), a total of 2,971 single nucleotide polymorphisms (SNP) are annotated in dbSNP (build 130) which we address, more appropriately, as multisite variations (MSV) [31].

A potential method to assess individual haplotypes at such loci is the amplification of an MSV-containing region by PCR [16]. Subsequently, the amplicons are subcloned in plasmids, individual bacterial clones are Sanger sequenced and haplotypes are determined according to the bases at the polymorphic positions. This approach, however, has inherent problems. First, individual CNs can vary between 2 and 12 DEFB copies per diploid genome. Theoretically, each genotype can comprise a number of haplotypes equal to this CN.

For individuals with many copies there is a high risk of missing rare haplotypes due to the decreased per-copy sequencing depth of 100-200 reads per genotype in a typical cloning/Sanger sequencing approach.

Second, PCR on complex templates may result in chimera formation by PCR-mediated recombination, creating artificial, false-positive haplotypes [32]. Third, the bacterial cloning step may introduce a systematic bias in the observed haplotype frequencies due to differences in the compatibility of clones with the host's lifecycle. To minimize the risk of spurious haplotyping by PCR/subcloning, independent PCR products have to be analyzed and large numbers of clones should be sequenced. This necessary quality control is usually too costly and labour intensive for high-throughput applications. In addition to assessing sequence variations, haplotyping has also been used for the measurement of DEFB CNs [16,17]. Absolute CNs, however, cannot be determined and even reliable relative CN measurement by this method is challenging. E. g. in the simplest case of just 2 copies and 2 haplotypes in an MSV spanning region, relatively small numerical deviations from the theoretical ratio of 1.0 (1:1) may result in quite different minimal CN estimations, as 1.2 (6:5) indicates 11 copies, 1.25 (5:4) indicates 9 and 1.33 (4:3) indicates 7.

For effective and accurate determinations of absolute DEFB CN, multiplex ligation-dependent probe amplification (MLPA) [28,33], real-time PCR [26,34,35], multiplex amplifiable probe hybridisation combined with restriction enzyme digest variant ratios (MAPH/REDVR) [36] and different variants of paralog ratio tests (PRT) [18,28,36] have been applied, however, they do not provide haplotype information. Knowledge, however, of both DEFB CN [20,29,30] and haplotypes [18] is of great importance with respect to clinically important phenotypes. Therefore, an ideal method for DEFB analysis and association studies would provide both CNs and haplotypes at once.

Application of massively parallel 454 technology to PCR amplicons allows extreme in-depth sequencing resulting from the huge amount of data generated by a single instrument run. Given a 250 bp amplicon and assuming 12,000 reads of ~250 bp length per 1/16^th ^segment of a GS FLX sequencer picotiterplate (GS FLX amplicon protocol), 16 PCR products can be sequenced in parallel with 12,000× coverage. This over-sequencing can be reasonably reduced using a multiplex approach, pooling either different amplicons generated from the same DNA [37,38] or the same amplicons derived from multiple DNAs using a barcoding strategy [39]. However, in the example above, pooling of 8 amplicons should still lead to a mean coverage of 1,500. This amount of sequences per individual amplicon is still sufficient to reduce the probability for haplotyping errors as described above. Furthermore, 454 amplicon sequencing does not require bacterial cloning, avoiding the danger of respective biases. As result, it delivers a multiple of individual sequences for a given amplicon compared to a routine cloning/Sanger sequencing strategy.

We took advantage of this methodological approach for DEFB haplotyping and CN estimation by 454 sequencing to study the DEFB locus in 11 DNA samples from publicly available lymphoblastoid cell lines (LCL). The results are compared to those obtained by the cloning/Sanger approach, and to CN measurements using MLPA, MAPH/REDVR and PRT.

## Methods

### DNA

Genomic DNA from the CEPH Resources (NA12760), the DNA Polymorphism Discovery Resource Collection (NA15029, NA15213, and NA15385) and the [40] (NA18502, NA18552, NA18858, NA19140) were purchased at the Coriell cell depository http://ccr.coriell.org/. DNAs C0140, C0766 and C0913 were obtained from the European Collection of Cell Cultures ECACC [41]. All 11 DNAs are derived from human LCLs, which were established from individuals of different ancestry, i.e. Caucasian (C0140, C0766, C0913, and NA12760), Yoruba (NA18502, NA18858, and NA19140) and Han Chinese (NA18552). For NA15029, NA15213, and NA15385 no information about the ethnicity of the donors is provided by the Discovery Resource Collection.

### 454 amplicon sequencing

PCR methods have been established to generate 8 DEFB specific amplicons (F1 to F8) using 454 adaptors A and B containing fusion primers (HPLC purified, TIBMolBio; Additional file 1, Table S1). PCRs were carried out with 50 ng DNA using the "Advantage" mixed polymerase containing both *Taq *and a proofreading enzyme (Clontech 639201) and 35 cycles of 94°C for 30 s, 57°C for 1 min and 72°C for 2 min followed by 72°C for 10 min. Concentrations of the PCR products were measured by Nanodrop and the Quant-IT^® ^PicoGreen^® ^ds DNA assay (Invitrogen). All amplicons derived from an individual DNA sample were mixed in an equimolar ratio (~4 × 10^10 ^molecules per amplicon). The pools were diluted and subjected to emulsion PCR following the FLX emPCR protocol for amplicons (Roche Diagnostics, December 2007) using both emPCR kits II (primer A) and III (primer B) and sequenced on a GS FLX (Roche Diagnostics) by both primers on 1 lane/pool of a 16-lane gasket on a 70 × 75 FLX picotiterplate. The sequences from 1 lane were aligned to a backbone consisting of the merged PCR target regions derived from the human reference sequence (NCBI build 36.1, hg18, proximal gene cluster) and haplotype calls (HCs) were inferred using the GS Amplicon Variant Analyzer Software (Roche Diagnostics).

For verification, amplicon F5 (*DEFB4*) was generated from NA12760 and NA18502 by a separate PCR using the same fusion primers and conditions as described for the amplicon pools. GS FLX sequencing was done on 2 lanes/amplicon of a 16-lane gasket on a 70 × 75 picotiterplate. To further increase sequencing depth, a third GS FLX run was performed with pooled amplicons F2, F4, F6 and F8 of NA12760, NA18502, NA18552, and NA18858 using a 4-lane gasket on a 70 × 75 picotiterplate. To avoid misinterpretations caused by incidental chimera formation early in PCR, 4 independent reactions were carried out with each primer pair and DNA sample.

### Amplicon subcloning in bacteria and Sanger sequencing

Using 7 out of the 11 DNAs (C0140, C0766, C0913, NA12760, NA18502, NA18858, NA19140), 2 PCR products (S1 and S2 [16]) were generated using unmodified primers (Metabion, Additional file 1, Table S1). PCRs were performed with 50 ng DNA using *Taq *polymerase (BIORAD). Amplification was achieved using 5 cycles of 95°C for 30 s, 56°C for 30 s and 72°C for 1 min followed by 30 cycles of 95°C for 30 s, 58°C for 30 s and 72°C for 1 min, and finally 72°C for 5 min. PCR products were subcloned into PCR2.1-TOPO vector (Invitrogen) and 192 individual clones per DNA and amplicon were sequenced by dye terminator sequencing chemistry using an ABI3730xl automated sequencer (Applied Biosystems) and M13rev universal primers. Haplotypes were inferred by manual inspection of Phrap alignments using GAP4 (Staden package).

### Other copy number measurement methods

The pyrosequencing based PRT (PPRT), the MLPA analyses [28] and the 5-PRT [18] were carried out as previously described. Results of CNs measured by MAPH/REDVR and another PRT [36] were kindly provided by J. Armour and E. Hollox.

## Results

### Haplotyping by 454 amplicon sequencing and comparison with the cloning/Sanger approach

The amplicons used in the present study were designed in order to cover a broad spectrum of regions within DEFB encompassing putative promoters, exons and introns and intergenic parts. If possible, amplicon lengths were restricted to span not much more than 250 bp in order to obtain consistent haplotype information by 454 reads from both ends. Furthermore, the amplified regions were selected to contain a high number of known MSVs. Application of these criteria resulted in 8 amplicons (F1 to F8) with lengths of 194-340 bp, spread over a region of 87 kb (Fig. 1) and harbouring 32 known MSVs (31 single nucleotide exchanges and one single base insertion/deletion, dbSNP build 130, Additional file 1, Table S2). Each amplicon harbours between 2 (F2) and 6 MSVs (F8). Eleven genomic DNAs were selected to represent a broad range of CNs as determined by MLPA, that is, 2-9 DEFB copies per diploid genome. PCRs were performed separately for each DNA and primer pair followed by equimolar pooling of all PCR products of the same DNA. For validation purposes, 2 additional amplicons from 7 samples were analysed by cloning/Sanger sequencing [16] (S1: 4 MSVs/500 bp, S2: 5 MSVs/529 bp; Fig. 1, Additional file 1, Table S2).

Eleven lanes of a single GS FLX run (one amplicon pool per 1/16^th ^plate) resulted in 145,836 reads with an average length of 225 bp, producing a total of 32.8 Mb raw data. Of the total reads, 142,033 reads (97.4%) could be assembled to the amplicon reference sequences resulting in an average of 12,912 reads per DNA sample and 18,230 per amplicon (Additional file 1, Table S3). Among the amplicons, F1 and F3 were underrepresented by 6,955 and 8,082 reads, respectively, compared to 15,003-24,723 for the remaining 6 amplicons. Additionally, the amount of reads per DNA sample greatly differed for F1 and F3 with less than 100 reads in 4 cases. Therefore, we have excluded the data of F1 and F3, leaving a total of 126,996 reads from 6 amplicons used for further analyses of the 11 DNA samples.

Every read may be regarded as representation of a particular DEFB allele. A haplotype can be inferred from each read by determination of the MSV allele combination, named "haplotype call" (HC). As amplicons were designed in accordance with the read length achievable by the GS FLX chemistry (~250 bp), the small minority of reads greatly shorter than 250 bp produced by the FLX sequencing may not span the aspired haplotype. Such shorter HCs, however, are less informative and would complicate the analyses. Therefore, these suboptimal HCs were omitted from further analyses, reducing the final data set from 126,996 reads to 123,003 most informative HCs (Additional file 1, Table S4).

Out of the 24 known MSVs within the 6 amplicons, 23 were found to be polymorphic. For rs4840825 (MSV16, amplicon F7), exclusively the T allele was observed. In addition, we confirmed in amplicon F5 of C0140, NA18858 and NA19140 an MSV at position 31 downstream of *DEFB4 *(A/G; chr8:7,259,758 and 7,791,677; MSV9a) not annotated in dbSNP but present in the hg18 reference sequence as a paralogous sequence variation [31]. Moreover, we identified a novel base exchange in the same amplicon of NA18502 located 24 nucleotides further downstream (C/A; chr8: 7,259,734 and 7,791,701; MSV10a).

The MSV combinations in 5 out of 6 amplicons could be unambiguously deduced from the overlapping forward and reverse reads. However, for F5 (340 bp, MSVs 6-10a) the read length of the FLX chemistry was too short to directly infer complete haplotypes, as the forward reads cover MSVs 6-8 and the reverse reads 7-10a, respectively (Table 1). Nevertheless, using data from non-polymorphic samples, the HC ratios in polymorphic samples and a parsimony assumption, 4 forward and 7 reverse haplotypes collapse consistently into 7 complete F5 haplotypes (Additional file 1, Table S5).

**Table 1:** Haplotype calls from 454 (run 1) and cloning/Sanger (CS) sequencing.

Method	454		CS	454		CS	454		CS	454		454		454		454		454	CS
Amplicon	F2		S1	F5		S2	F5		S2	F4		F6		F7		F8		Total	
MSV HCs	1-2	#	#	6-8	#	#	7-10a*	#	#	3-5	#	11-15	#	16-18	#	19-24	#	HCs	#
**C0140**	GA	1.566	112	TCC	664	136	CCGgGc	1.491	81	TAT	582	CCTCG	867	TCG	888	CTACCG	542		
	GG	583	36				CCGaGc	431	55	CAT	390	CCTAG	816	TTA	288	TCTTTA	195		
	CA	528	30							TAC	382							**10.213**	**450**

**C0766**	GG	1.525	114	TCC	360	140	CCGgGc	1.084	147	TAC	1.679		nd	TCG	1.878	CTACCG	876		
	CA	842	41	CCC	195	48	CCGgAc	506	41	CAT	601							**9.546**	**531**

**C0913**	CA	1.869	113	TCC	680	132	CCGgGc	1.221	145	TAC	1.556	CCTCG	1.195	TCG	1.816	CTACCG	2.153		
	GA	1.157	58	CCC	287	54	CCGgAc	617	41	TCC	634	CCTAG	593					**13.778**	**543**

**NA12760**	GA	1.750	92	TCC	795	99	CCGgGc	1.566	104	TAC	2.825	CCTCG	1.669	TTA	2.056	TCTTTA	645		
	GG	386	20	CCC	181	60	CCGgAc	339	55	TAT	981	CTCAC	599	TCG	1.696	CTACCG	605		
	CA	319	19	CTG	184	0	TGGgAc	459	0	TCC	797	CCTAG	499					**18.351**	**449**

**NA15029**	GA	1.530		TCC	767	nd	CCGgGc	1.046	nd	TCC	1.156	CCTGC	1.785	TTA	1.689	TCTTTA	377		
	GG	805					CCAgGc	431		TAT	882	CTCAT	1.087	TCG	1.115	CTACCG	267		
				CTG	475		TGGgAc	913		TAC	496								
										CAT	420							**15.241**	

**NA15213**	GA	1.326		TCC	665	nd	CCGgGc	1.338	nd	TAC	278	CCTCG	1.178	TCG	1.496	CTACCG	929		
	CA	349		CTG	171		TGGgAc	390		TAT	98	CCTAG	577	TTA	866	TCTTTA	551		
										TCC	87	CTCAT	473					**10.772**	

**NA15385**	GA	985		TCC	374	nd	CCGgGc	702	nd	TAC	1.374	CCTCG	1.003	TCG	938	CTACCG	688		
	GG	592		CCC	169		CCGgAc	239		TAT	673	CCTAG	322	TTA	782	TCTTTA	483		
				CTG	88		TGGgAc	188		TCC	345	CTCAT	280					**10.225**	

**NA18502**	GA	812	80	TCC	182	125	CCGgGc	265	139	TAT	1.453	CCTCG	415	TTA	856	TCTTTA	645		
	CA	561	58	CCC	21	15	CCGgGa	56	0			TTCAT	65	TCG	418	CTACCG	267		
				CCG	(7)	0	CGGgAc	39	0									**5.995**	**402**

**NA18552**	CA	3.490		TCC	197	nd	CCGgGc	273	nd	TAT	225	CCTCG	1.653	TCG	1.756	CTACCG	1.525		
				CCC	328		CCGgAc	340		TAC	1.058								
										CAT	503							**11.348**	

**NA18858**	GA	677	97	TCC	1.234	188	CCGgGc	623	124	TAT	1.490	CCTCG	1.327	TTA	1.087	TCTTTA	2.404		
	CA	324	45				CCGaGc	218	51	TAC	822			TCG	404	CTACCG	633		
	GG	205	(16)															**11.448**	**505**

**NA19140**	GA	314	57	TCC	706	nd	CCGgGc	234	nd		nd	CCTCG	504	TTA	281		nd		
	CA	310	73				CCGaGc	42				CTCAT	130	TCG	62				
	GG	105	24															**2.688**	**154**

**Total**		22.910	1.069		8.702	982		15.012	983		21.787		17.037		20.372		13.785	**119.605**	**3.034**

#: number of HCs; *: alleles in lower cases refer to the 2 additionally identified MSVs 9a and 10a; (): nominally below 10% threshold; nd: no data.

**Figure 2:**
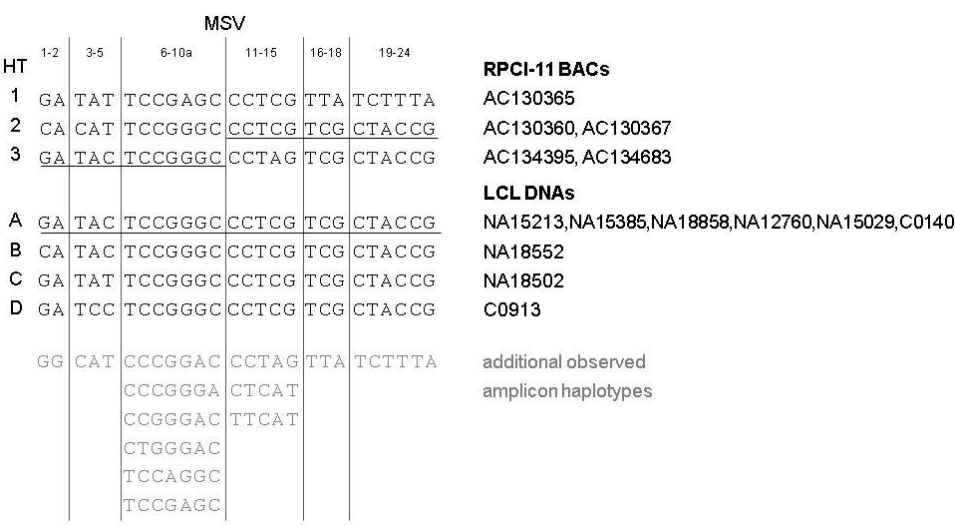
**Comparison of physical long range DEFB haplotypes derived from RPCI-11 Human Male BAC sequences (HT 1-3) and HC inferred long range haplotypes (HT A-D)**. Underline: relation of haplotypes 2, 3 and A; gray: additional observed amplicon haplotypes indicating that all BAC specific patterns are also present in the 11 analyzed samples.

In total, 22 haplotypes could be identified (Table 1, Additional file 1, Table S5). The largest variety was found for amplicon F5 with 7 haplotypes out of 128 theoretically possible combinations of MSVs. In contrast, only 2 haplotypes were observed for F7 (8 theoretical haplotypes) and F8 (64), respectively. The most abundant haplotypes are for F2: GA, F4: TAC, F5: TCCGGGC, F6: CCTCG, F7: TCG and F8: CTACCG,. TCCGGGC and TCG were found in all investigated DNAs. Joining the haplotypes with the most HCs for each DNA sample individually creates 4 inferred "long range haplotypes" of which one is present in of 6 samples (Additional file 1, Table S6). Remarkably, this inferred haplotype can be generated by just one recombination of physical haplotypes represented by large insert clones from the RPCI-11 Human Male BAC Library, sequenced in the course of the human genome project [16] (Fig. 2).

Several additional MSV combinations were found which are represented by only few reads compared to the haplotypes described above, indicating either the generation of chimeric, PCR-mediated recombinant molecules from different target copies, misincorporations during the emulsion PCR, sequencing errors or cross-contaminations. In total, 53 out of 66 454 PCRs and 10 out of 22 Sanger PCRs contained this phenomenon. Based on the estimation by alternative methods that the maximal DEFB CN in our sample set is 9, we discarded HCs with fractions <0.10 (1/10) as such artefacts. This led to the exclusion of 3,338 (2.7%) and 86 (2.7%) HCs from the 454 and Sanger datasets, respectively (Table 1, Additional file 1, Table S4).

Comparing the haplotyping results for both methods, the same haplotypes were found in 85% (11/13) except for amplicons F5/S2 in samples NA12760 and NA18502 (Table 1). In both cases rare 454 haplotypes were not found in the Sanger reads. Due to the low overall number of F5 sequences for NA18502, the HC frequency for the haplotype inferred from MSVs 6-8 is lower <0.10 and had to be excluded (Table 1, value in brackets; Additional file 1, Table S4). In order to finally confirm or reject the questionable haplotypes, we generated additional F5 amplicons from the 2 DNA samples and performed 454 sequencing in 2/16^th ^plate per sample resulting in 33,305 and 33,205 sequences, respectively. From these reads more than 10,000 HCs per sample for MSVs 6-8 and over 20,000 calls for MSVs 7-10a were obtained for each of the 2 DNAs, unequivocally confirming the presence of the questionable CTGGGAC and CCGGGAC haplotypes, respectively (Table 2).

**Table 2:** Haplotype calls of ultra-deep amplicon F5 454 sequencing (run 2) for NA12760 and NA18502.

	**F5 forward**			**F5 reverse**		
**MSV HCs**	**6-8**	**#**	**f**	7-10a*	**#**	**f**
	
**NA12760**	TCC	7.399	0,69	CCGgGc	14.683	0,72
	CCC	1.765	0,16	CCGgAc	2.891	0,14
	CTG	1.591	0,15	TGGgAc	2.895	0,14
Total		**10.755**			**20.469**	
	
**NA18502**	TCC	8.307	0,80	CCGgGc	17.586	0,83
	CCC	1.069	0,10	CCGgGa	1.764	0,08
	CCG	1.006	0,10	CGGgAc	1.950	0,09
Total		**10.382**			**21.300**	

#: number of HCs; f = HC frequency; *: alleles in lower cases refer to the 2 additionally identified MSVs 9a and 10a.

### Determination of relative DEFB copy numbers by haplotype calls

In order to evaluate the applicability of 454 HCs for the measurement of relative DEFB CNs, we calculated the HC ratios for all amplicons with more than one haplotype and inferred the respective minimal CN. For example, in F2 from C0140, 3 haplotypes (CA, GA, and GG) were identified with 528, 1,566 and 583 calls, respectively. This corresponds to a normalized call ratio of 1:2.97:1.10 that is essentially 1:3:1, indicating a minimal CN of 5. Altogether, by this approach 60 CN calculations were done. For samples with more than one polymorphic amplicon we computed the average of the separate CNs or of their least common multiple. The final minimal CN estimations for the samples range between 3 and 9 (Table 3, Additional file 1, Table S7).

**Table 3:** DEFB copy numbers per diploid genome estimated by the ratio of 454 haplotype calls (run 1) in comparison to alternative methods.

	454								Alternative methods							
Amplicon	F2	**F5**f	**F5**r	F4	F6	F7	F8	Average		Armour	5PRT	PPRT	MLPA	Average		**454-Altern**.
MSVs	1-2	6-8	7-10a	3-5	11-15	16-18	19-24		~						~	~
**C0140**	5		4		4 (2n)	4	4	4.20	**4**	4	3.78	4.19	4	3.99	**4**	**0**
**C0766**	3	3	3	4				3.25	**3**	3	2.75	3.31	3	3.01	**3**	**0**
**C0913**	3	3	3	3	3			3.00	**3**	3	3.00	3.13	3	3.03	**3**	**0**
**NA12760**	7	6	6	6	5	6 (2n)	6 (2n)	6.00	**6**	6	5.57	6.04	6	5.90	**6**	**0**
**NA15029**	6 (3n)	5	5	6	5	5	5	5.29	**5**	nd	4.70	5.58	5	5.09	**5**	**0**
**NA15213**	5	5	4	5	4	5	5	4.71	**5**	nd	4.18	4.18	4	4.12	**4**	**+1**
**NA15385**	5	7	5	7	5	6 (2n)	5	5.71	**6**	nd	5.29	5.17	5	5.15	**5**	**+1**
**NA18502**	10	10	9		7	9 (3n)	8 (4n)	8.83	**9**	8	8.98	nd	9	8.66	**9**	**0**
**NA18552**		5	6 (2n)	7				6.00	**6**	2	2.23	2.02	2	2.06	**2**	**+4**
**NA18858**	4		4	3		4	5	4.00	**4**	8	7.71	8.08	8	7.95	**8**	**-4**
**NA19140**	7		7		5	6		6.25	**6**	7	5.71	6.26	6	6.24	**6**	**0**

Armour: PRT and MAPH/REDVR [36] and personal communication; 5PRT. PPRT [18,28]; MLPA [28]. ~: rounded; n: multiple of the minimal estimated CN; *: CN estimations based on ultra-deep 454 sequencing; nd: no data; blank: non polymorphic.

The DEFB CNs of the 11 samples haplotyped in this study were previously measured by 40 independent experiments using different PRTs, MLPA and MAPH/REDVR [18,28,36] and Armour and Hollox, personal communication]. For 8 of the 11 DNAs, measurement by the different techniques (not including our) resulted in identical CNs. The remaining samples showed disagreements of only one copy, indicating the consistency of the data (Table 3, Additional file 1, Table S7). Therefore, we took the average of these measurements and compared them with CN estimates from the 454 HCs. For 7 samples the 454 haplotype-based estimates were in agreement with CNs determined by the alternative methods. For another 2 DNA samples (NA15213, NA15385), these numbers differed only by one copy. In the case of NA18858, the estimated minimal CN was half of the absolute one. For NA18552, 3 MSV combinations are polymorphic, resulting in CNs of 5 (2:3), 2n (1:1) and 7 (2:4:1), respectively. Only in this single case, the HC-based minimal CN estimations can not be unambiguously interpreted and considerably deviates from 2 copies per diploid genome, consistently determined by 4 alternative methods.

As ultra-deep sequencing of NA12760 and NA18502 amplicon F5 provided most consistent CN estimations (Table 2, Additional file 1, Table S7) for samples with high CNs, we further validated this approach by performing a third GS FLX run with pooled amplicons F2, F4, F6 and F8 from these DNAs. Moreover, we included NA18552 (CN = 7 by HCs of amplicons F4) and NA18858 (CN = 8 by alternative methods) (Table 3). To avoid false positive HCs resulting from chimera formation early in PCR, we carried out 4 independent reactions with each primer pair and DNA sample. Accordingly, we lowered the threshold for exclusion of minor haplotype fractions from 10% to 6% to avoid false negative HCs. Altogether, we obtained 405,574 HCs providing on average ~25,000 per amplicon and sample (Additional file 1, Table S8), of which 1.4% were excluded as potential chimera, sequencing errors or contaminations. The extraordinary sequencing depth and the lowered filter threshold identified additional haplotypes for NA18502 (F2: GG) and NA18858 (F4: CAT, F6: TTCAT) which increased the resolution of the CN estimation (Table 4, Additional file 1, Table S9). Ultra-deep sequencing-based CN estimations for samples with high CNs (NA18502, NA18858) tend to be higher than those determined by the established alternative methods.

**Table 4:** DEFB copy numbers per diploid genome estimated by 454 ultra-deep sequencing (run 2: F5; run 3: F2, F4, F6, F8) in comparison to alternative methods.

	454							Alternative methods	**454-Altern**.
Amplicon	F2	**F5**f	**F5**r	F4	F6	F8	Average	Average	
**NA12760**	8	7	7	7	5	9	**7**	6	**+1**
**NA18502**	11	10	11		9	10	**10**	9	**+1**
**NA18552**		nd	nd	8			**8**	2	**+6**
**NA18858**	10	nd	nd	12	11	10	**11**	8	**+3**

nd: no data; blank: non polymorphic.

## Discussion

Impaired beta-defensin synthesis has been described in many human diseased states, namely in inflammatory disorders [20,22,29,30,42-44]. While association of a disease and DEFB CN has been demonstrated [20,29,30], associations of sequence variants with disease [18] cannot reliably be investigated with established genotyping methods. Hollox [22] observed that it may be the nucleotide state of an MSV that actually causes susceptibility to a disease for which association with CN has been found. If so, CN is only a proxy for causative sequence variants. However, description of sequence variation in CN variable loci such as DEFB at 8p23 is inherently challenging. Currently, neither the haplotype structure of DEFBs nor their arrangements on chromosomes (DEFB-locus alleles) are known. The present work provides a first glimpse of the DEFB haplotype complexity through 454 amplicon sequencing of selected polymorphic defensin gene fragments. Among the currently available next generation sequencing (NGS) approaches, the 454 technology is best suited for this purpose as it provides longest reads that in turn produce the longest inferred physical haplotypes.

Eleven DNA samples derived from LCLs were selected to represent a range of 2-9 DEFB copies per diploid genome. All DNAs are publicly available and 4 were investigated in the framework of the HapMap project [40]. Four of the LCLs were established from individuals of Caucasian, 3 of Yoruba and one of Chinese ancestry. About 120,000 MSV HCs were derived from 6 amplicons containing 2-6 annotated MSVs each. One MSV not deposited in dbSNP and a new one were detected in 3 samples of Yoruba and one of Caucasian ancestry, respectively. Theoretically, the 26 MSVs in 6 amplicons altogether could compose 268 haplotypes of which we identified 22 (8%). As exemplified by the analysis of the F5 amplicon data, HC ratios provided by the 454 deep sequencing approach may serve as additional experimental evidence to deduce longer haplotypes than the maximal NGS read length.

With 26 MSVs in 6 amplicons, totalling to 1,498 bp, the MSV density in the investigated regions is approximately 17 MSV/kb. This is higher than the overall single nucleotide variation density of the entire DEFB currently annotated in dbSNP (2,971 MSVs/229 kb = 13 MSV/kb) and reflects the directed selection of the amplified regions to screen a maximum of polymorphic positions within a short distance. In comparison, for the most sequence variable locus of the human genome, the MHC complex, alignments of 8 MHC haplotypes revealed 8 SNP/kb (37,451 SNPs/4.7 Mb) with a higher density (13 SNP/kb) in the intragenic regions [45]. For the genomic loci of HLA-A, B and C there are 1,578 polymorphic positions within 10,020 bp assigned by dbSNP build 130, corresponding to 157 SNPs/kb. This indicates that in terms of sequence variability, DEFB is substantially less polymorphic than the HLA loci, and that DEFB together with the analysed amplicons are comparable to the MHC intragenic regions. The fact that the 454 deep-sequencing revealed only one novel MSV on the background of 24 previously known ones shows that the dbSNP annotation of the analysed regions is quite comprehensive. On the other hand according to dbSNP, the MSV density of DEFB is about 4 times higher than the SNP density of the entire human genome (4 SNP/kb). Moreover, currently unknown additional sequence variability may be still hidden in the DEFB (e.g. 25 MSV/kb upstream of *DEFB4 *observed in 16 LCLs; Groth, unpublished) which may be revealed by future targeted and whole-genome resequencing efforts.

The haplotypes identified by 454 sequencing and by cloning/Sanger sequencing were identical except for 2 cases, which could be resolved by additional 454 sequencing. However, the ratios of haplotypes derived from a given individual amplicon sometimes differ remarkably. For example, the C0913 F5 haplotypes CCGGAC and CCCGGC were revealed by 454 sequencing in a ratio of 1:2 (617/1,221 calls) but 1:3.5 (41/145 calls) by Sanger derived reads. We suspect the explanation is that the number of HCs from Sanger sequenced clones is too low for providing reliable results. Moreover, additional bias may be introduced e.g. by the bacterial cloning step.

Theoretically, the more HCs that are available, the more accurately they should reflect the real ratio of the DEFB copies with particular haplotypes. This becomes more important as the number of DEFBs and/or haplotypes per genome increases. The conclusion is supported by our efforts to resolve the discrepancies between 454 and Sanger sequencing for NA12760 and NA18502. The minor haplotypes of the F5 amplicon were supported by 454 but not by Sanger sequencing, probably due to the low amount of sequenced clones. Additional 454 HCs finally confirmed the presence of the F5 haplotypes under question.

**Figure 3:**
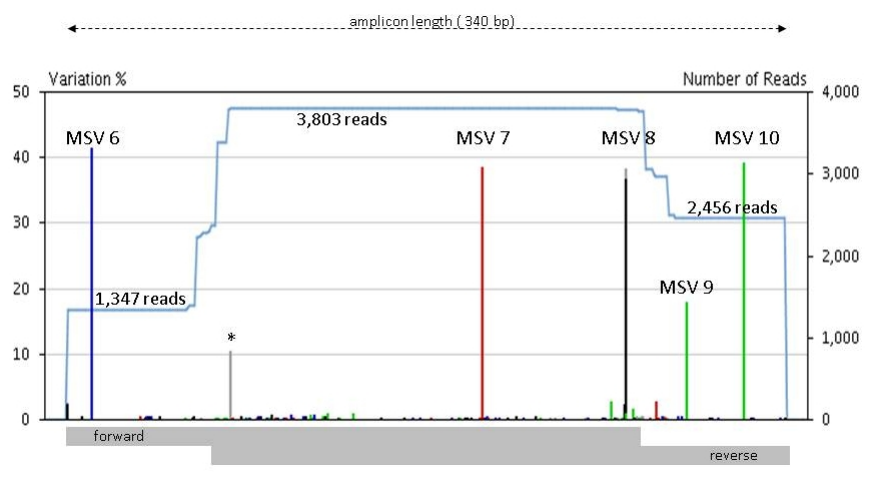
**Graphical output of the GS Amplicon Variant Analyzer for the assembly of sequences as exemplarily shown for NA15029, amplicon F5**. Left Y-axis: minor allele frequency; MSV colour code: blue = C; red = T; black = G; green = A; asterisk: insertion/deletion 454 sequencing artefact within a poly-A stretch; blue curve: read coverage; grey bar below: average lengths of forward and reverse reads, respectively.

Furthermore we noticed an uneven distribution of forward and reverse reads in the 454 sequencing of particular amplicons. This is obvious in Fig. 3 with a nearly 1:2 forward-to-reverse read ratio and is also reflected in the entire data set of amplicon F5 with 8,702 forward but 15,020 reverse HCs (1:1.7). Such a skew may be caused by unequal sequencing efficiencies due to the direction-specific sequencing primers as well as to differences in base composition and/or motifs between the forward and reverse strands. Thus in the case of non or partially overlapping forward and reverse reads, the resolution of the approach is limited by the less effective sequencing direction [46,47].

Another problem of any PCR-based haplotyping is the occurrence of chimeric products [32]. Since both 454 amplicon sequencing and the cloning/Sanger approach are based on PCR, the formation of such artefacts must be taken into account. Chimeras can be generated during PCR by heteroduplexes of e.g. a complete copy of one allele with a fragment from another allele. Completion of the partial strand in the elongation step creates chimeric molecules from which chimeric haplotypes may be erroneously determined. The degree of chimera formation is dependent on various factors such as polymerase, nucleotide/primer concentrations, cycling conditions and amplicon length. In addition to the formation of chimeric PCR products, nucleotide misincorporations during the emulsion PCR, sequencing errors and cross-contaminations have to be taken into account. In the current study, we have appointed and discarded an MSV combination as artificial if its relative number of call was lower than 10% (1/10; cloning/Sanger and GS FLX run 1) or 6% (1/16; ultra-deep FLX runs 2 and 3). This led to the exclusion of less than 3% of all HCs which otherwise would render the analysis impossible. False negative HCs by this cutoff *per definitionem *would only affect DNA samples with more than 10 and 16 DEFB copies, respectively. CNs >10 are rarely reported in the literature [23,29,30]. Nevertheless, these threshold filters may fail if the artificial molecule appears early in PCR. To avoid respective misinterpretations, independent PCRs were carried out and pooled before sequencing for the third GS FLX run.

The estimation of CNs from the HC ratios is intrinsically problematic, since ratios provide only minimal CNs and any multiple of that can be ruled out only by additional considerations (NA18858). Moreover, slightly different ratios lead to different CN estimations. Nevertheless, thanks to the high number of NGS HCs, CNs could be correctly determined for 7 out of 11 samples and differed only by one copy per diploid genome for another 2 samples (Table 3, Additional file 1, Table S7). Remarkably, the high HC numbers obtained by ultra-deep GS FLX runs 2 and 3 consistently point towards higher CNs than obtained by the widely used PRTs/MPLA (Tab. 4, Additional file 1, Table S9). It is known that the latter methods tend to underestimate the actual CN in case of DEFB CNs >5 (Huse, unpublished). Thus, it has to be proven in the future, whether ultra-deep NGS like 454 may provide more reliable results than the currently established methods for cases of high CNs per diploid genome. The recently developed mrFAST (micro-read fast alignment search tool) [48] points in the same direction although the sequencing depth of personalized whole-genome sequencing efforts and the 1000 Genomes Project is too low to provide sufficient read counts for reliable estimations of high CNs in complex regions like DEFB.

Only in one case were the CN estimations inconsistent (NA18552: 5-8 copies by 454 sequencing *vs*. 2 copies by 4 alternative methods, Tables 3 and 4). For this, we suppose 2 possible reasons. First, the estimated CN 5 results from amplicon F5 with a relatively low amount of reads which in case of the forward sequencing direction (MSVs 6-8) caused a random deviation from the 1:1 ratio observed for the reverse HCs. Second, 7-8 copies were calculated by the ratio of 3 haplotypes in amplicon F4. As the 3 haplotypes were confirmed by ultra-deep sequencing of pooled independent amplicons, it is unlikely that the minor haplotype (TAT) is a chimera. As the remaining 454 amplicons show no sequence variation and are therefore in agreement with the low CN of 2 determined by the alternative methods, sample NA18552 may represent in our hands the first example violating the concordance rule of DEFB [28]. Whether the underlying molecular event represents a natural polymorphism or emerged in cell culture remains to be elucidated in the future.

Although our results indicate that NGS based haplotyping and CN estimation thereof is a suitable approach to characterize a highly polymorphic and CN variable loci as the DEFB cluster, this method has some inherent limitations. First, haplotyping is restricted to those regions in which at least two MSVs are located within the read length of the applied NGS technology. Second, equimolar pooling of PCR products is difficult and also the efficiencies of the following emulsion PCR may deviate among the amplicons. This can result in differing amounts of HCs per amplicon which may prevent reliable haplotyping if too few sequences are obtained (e.g. from our amplicons F1 and F3). Third, formation of PCR artefacts leading to the identification of "false", i.e. chimeric haplotypes always has to be considered. Although distinction between "true" and "false" halpotypes is easier with higher sequencing depth it is problematic for samples with high CNs.

## Conclusion

Deep 454 amplicon sequencing is an effective method for parallel haplotyping and CN estimation in highly polymorphic loci such as DEFB at chromosome 8p23.1 for the following reasons:

1. There is no bacterial cloning bias as a source of putative errors in haplotype identification. Moreover, in contrast to the cloning/Sanger sequencing approach, thousands of HCs per amplicon can easily be performed leading to a higher accuracy in obtained haplotype ratios and CN estimations inferred thereof.

2. Calculated on the basis of the consumable's prices for the present study and normalized to the same yield of informative HCs, the costs for 454 amplicon sequencing amounts to only ~4% of those for cloning/Sanger sequencing. Although this does not even consider the reduced experimental effort it is still too expensive for high-throughput measurements but acceptable for analyses of small cohorts.

3. Recently, 454 Titanium amplicon sequencing protocols and kits became available (Roche Diagnostics), increasing achievable read lengths to 400 bp with a 2-fold increase of the number of reads per run. Thus, longer amplicons than in our study can be sequenced, encompassing more or more distant MSVs. Moreover, as the output of reads also increases, multiplexing different DNAs will be feasible by application of barcoding strategies (multiplex identifier, MID).

4. Although the presented 454 amplicon sequencing delivers only relative CNs and has to be completed by other approaches, future inclusion of reference amplicons from loci with invariable CN will provide absolute DEFB CNs.

Deep 454 amplicon sequencing can contribute substantially to investigate sequence variants and haplotype structure of CN variable loci. Nevertheless, increases in both NGS read length and output are needed to eventually completely resolve loci like DEFB at the physical haplotype level.

## Authors' contributions

ST designed the 454 experiments and carried out both the 454 and Sanger experiments, designed by KH. MG performed the MLPA and PRT experiments. ST, AP and KS analyzed the data. MP, ST, JH, PR and SS conceived of the study, and participated in its design and coordination. ST, MP, KH and KS wrote the manuscript. All authors read and approved the final manuscript.

## Supplementary Material

Additional file 1 **Amplicons (F = GS FLX/S = Sanger sequencing), primers and their locations in the human genome**. PCR products (amplicons), primers used for their amplification and chromosomal locations (NCBI build 36.1, hg18) of the amplified regions. **MSVs and amplicons**. Analyzed multisite variations (MSVs), their rs numbers and chromosomal locations according to SNPdb build 130 and NCBI build 36.1 (hg18), respectively. **Numbers of assembled GS-FLX reads per amplicon (F1 to F8) and DNA (run1 and run 2)**. Assembled 454 sequences from 2 GS FLX runs per DNA and amplicon. **Haplotypes and haplotype calls (HCs) for GS-FLX runs 1-2 and PCR/cloning**. Haplotypes, haplotype calls (HCs) and their fraction per DNA and MSV combination, derived from GS FLX runs 1 and 2 and Sanger sequencing of cloned PCR products. **Haplotypes (HTs) by MSV combinations**. Haplotypes and total number and fraction of haplotype summed up from all DNAs sequenced in GS FLX runs 1 and 2. **Haplotypes by DNA**. Identified haplotypes per DNA and amplicon, highlighting the most abundant haplotypes. **Individual DEFB copy numbers (CN) derived from the ratio of haplotype calls (HCs) per MSV combination and comparison with CNs by other methods**. Estimation of of the DEFB cluster copy number (CN) from the ratio of haplotype calls (HCs) per DNA and amplicon and comparison of with CNs determined by MLPA and different paralogue ratio tests. **Haplotypes and haplotype calls (HCs) for GS-FLX run 3**. Haplotypes, haplotype calls (HCs) and their fraction per DNA and MSV combination, derived from GS FLX run 3 (ultra-deep sequencing). **Individual DEFB copy numbers (CN) derived from the ratio of haplotype calls (HCs) per MSV combination from run1+2 versus run3**. Individual DEFB copy numbers (CN) for 4 DNAs in comparison between GS FLX run 1+2 versus GS FLX run 3 (ultra deep sequencing). Click here for file
